# External validation of a prediction model and decision tree for sickness absence due to mental disorders

**DOI:** 10.1007/s00420-020-01548-z

**Published:** 2020-05-11

**Authors:** Marieke F. A. van Hoffen, Giny Norder, Jos W. R. Twisk, Corné A. M. Roelen

**Affiliations:** 1 Department of Research and Development, Human Total Care, Utrecht, The Netherlands; 2 Department of Epidemiology and Biostatistics, Amsterdam Public Health Research Institute, Amsterdam UMC, Location VU University Medical Center, Amsterdam, The Netherlands; 3 Department of Health Sciences, University of Groningen, University Medical Center Groningen, Groningen, The Netherlands; 4 HumanCapitalCare, Laan van Nieuw Oost-Indië 133-G, 2593 BM Den Haag, The Netherlands

**Keywords:** Health surveys, Mental health, Reproducibility of results, ROC analysis, Validation studies

## Abstract

**Purpose:**

A previously developed prediction model and decision tree were externally validated for their ability to identify occupational health survey participants at increased risk of long-term sickness absence (LTSA) due to mental disorders.

**Methods:**

The study population consisted of *N* = 3415 employees in mobility services who were invited in 2016 for an occupational health survey, consisting of an online questionnaire measuring the health status and working conditions, followed by a preventive consultation with an occupational health provider (OHP). The survey variables of the previously developed prediction model and decision tree were used for predicting mental LTSA (no = 0, yes = 1) at 1-year follow-up. Discrimination between survey participants with and without mental LTSA was investigated with the area under the receiver operating characteristic curve (AUC).

**Results:**

A total of *n* = 1736 (51%) non-sick-listed employees participated in the survey and 51 (3%) of them had mental LTSA during follow-up. The prediction model discriminated (AUC = 0.700; 95% CI 0.628–0.773) between participants with and without mental LTSA during follow-up. Discrimination by the decision tree (AUC = 0.671; 95% CI 0.589–0.753) did not differ significantly (*p* = 0.62) from discrimination by the prediction model.

**Conclusion:**

At external validation, the prediction model and the decision tree both poorly identified occupational health survey participants at increased risk of mental LTSA. OHPs could use the decision tree to determine if mental LTSA risk factors should be explored in the preventive consultation which follows after completing the survey questionnaire.

## Introduction

Mental disorders are the major cause of long-term sickness absence (LTSA) in member countries of the Organization of Economic Co-operation and Development (OECD 2015). Mental LTSA disconnects employees from the workplace and marginalizes them from the labor market, leading to unemployment, social isolation, and poorer mental health (Henderson et al. 2011). Therefore, it is important to identify employees at risk of mental LTSA before they report sick.

Previous studies have shown that distress symptoms identify non-sick-listed employees with an increased risk of future mental LTSA (Roelen et al. 2013; van Hoffen et al. 2015, 2016). Recently, Van Hoffen et al. (2019) included distress in a multivariable prediction model for mental LTSA with gender, marital status, economic sector, years employed at the company, role clarity, cognitive demands, learning opportunities, co-worker support, social support from family/friends, and work satisfaction as additional predictor variables. The authors reported that this 11-predictor model correctly assigned the highest risk to those who had mental LTSA during 1-year follow-up in 71.3% of the cases. Furthermore, they found that a 3-knot decision tree based on distress, gender, work satisfaction and work pace correctly identified employees with mental LTSA in 70.9% of the cases. Decision trees are easier to interpret than regression formulas and are therefore more user-friendly for healthcare practice (Loh 2014). However, decision trees more than regression models depend on the data in which they are developed, and may therefore be less valid in new populations of employees (Stiglic et al. 2012).

The aim of the present study was to validate the previously developed prediction model and decision tree in a new sample of occupational health survey participants. If externally valid, the prediction model and/or decision tree can be implemented in occupational healthcare practice to identify employees at risk of mental LTSA.

## Methods

### Study population and design

In The Netherlands, employers have to enable their employees to participate in occupational health surveys once every 4 years. Participation in occupational health surveys is voluntary for employees. Occupational health surveys are conducted by an occupational health service (OHS) and consist of an online survey questionnaire addressing health status and working conditions, followed by a preventive consultation with an occupational health provider (OHP, i.e., occupational physician or nurse). Preventive consultations as part of the occupational health survey are restricted to one session per participant. Those who need more support can be referred to other OHPs such as lifestyle coaches, physiotherapists, social workers, or psychologists.

In 2016, 3415 employees in mobility services were invited to participate in an occupational health survey. A total of 1736 (51%) non-sick-listed employees agreed to participate in the occupational health survey. The study was designed as a prospective cohort study with the occupational health survey as baseline and OHS recorded sickness absence as an outcome at 1-year follow-up. Results are presented in line with the transparent reporting of a multivariable prediction model for individual prognosis or diagnosis (Moons et al. 2015).

### Outcome: long-term sickness absence (LTSA)

Sickness absence was defined as a temporary paid leave from work due to any (i.e., work-related as well as non-work-related) injury or illness, and was recorded from the first to the last sickness absence day in the OHS register. In The Netherlands, sickness absence is medically certified by an occupational physician (OP) within 42 days of reporting sick. Therefore, LTSA was defined as sickness absence lasting 42 days or longer.

Based on a consultation with a sick-listed employee, the OP records a diagnostic code derived from the 10th International Classification of Diseases (ICD-10) in the OHS register. Mental LTSA was defined as LTSA with diagnostic codes of the ICD-10 chapter V (Mental and Behavioral Disorders). Mental LTSA in the 12 months prior to the occupational health survey was used for the predictor variable ‘prior mental LTSA’. Mental LTSA during 1-year follow-up was used as the outcome variable.

### Predictors

The predictor variables were measured with the same items and scales as in the development study (van Hoffen et al. 2019). Gender and the number of years employed at the company were retrieved from the occupational health survey questionnaire.

Work pace (five items, Cronbach’s *α* = 0.85), role clarity (five items, *α* = 0.84), cognitive demands (five items, *α* = 0.82), learning opportunities (four items, *α* = 0.86), and co-worker support (three items, *α* = 0.87) were measured with the Questionnaire on the Experience and Evaluation of Work (van Veldhoven et al. 2002). Survey participants responded on a five-point frequency scale ranging from ‘never’ (= 1) to ‘always’ (= 5) and item scores were summed to a total subscale score, which was then divided by the number of items in the scale. Consequently, all psychosocial work characteristics had a score range between 1 (= low) and 5 (= high).

Social support from family and friends was assessed with three items (Can you count on the support of partner/family/friends when you have some difficulty at work? Is work at home taken out of your hands if you are busier at work? Do you feel appreciated by your partner/family/friends?; *α* = 0.78). Survey participants responded on a five-point frequency scale ranging from ‘never’ (= 1) to ‘always’ (= 5) and item scores were summed and averaged so that social support from family/friends ranged between 1 (= low) and 5 (= high).

Work satisfaction was measured with six items (*α* = 0.91) about pleasure in work (e.g., I am pleased to start my day’s work; I find my work stimulating; I enjoy my work). Responses were given on 5-point frequency scales ranging from ‘never’ (= 1) to ‘always’ (= 5). Items scores were summed and averaged so that work satisfaction ranged between 1 (= low) and 5 (= high).

Distress was measured with the Four-Dimensional Symptom Questionnaire (4DSQ), which was included in the occupational health survey questionnaire. The distress scale consisted of 16 items addressing symptoms elicited by stressors or the efforts to maintain psychosocial functioning, such as worry, irritability, tension, listlessness, poor concentration, sleeping problems and demoralization (Terluin et al. 2004). Survey participants were asked if they experienced these symptoms in the past week, ‘no’ (= 0), ‘sometimes’ (= 1), ‘regularly’ (= 2), ‘often’ (= 2), or ‘very often/constantly’ (= 2). Item scores were summed (score range 0–32; Cronbach’s *α* = 0.94) so that higher scores reflected higher levels of distress. Terluin et al. (2008) defined scores ≤ 10 as low, 11–20 as moderate, and > 20 as high distress.

### Statistical analysis

Statistical analyses were done in IBM SPSS Statistics for Windows, version 24 (released 2016; IBM Corp. Armonk, NY).

#### Missing data

Of the 1736 occupational health survey participants, 116 (7%) had missing data on role clarity and social support from family/friends. The missing data were imputed in SPSS by using series means. Marital status was not available from the occupational health survey questionnaire and was therefore excluded from the prediction model.

#### External validation of the regression model.

The regression coefficients of gender, years employed at the company, role clarity, cognitive demands, learning opportunities, co-worker support, social support from family/friends, work satisfaction, and distress from the development setting were combined with the predictor values of the validation setting. As all employees worked in mobility services, the economic sector was constant.

#### External validation of the decision tree

The decision tree was based on the development study, using distress categories (low, moderate, high), gender, work satisfaction and work pace as predictor variables. In accordance with the development study, work satisfaction was dichotomized into low (≤ 3.3) and high (> 3.3). Likewise, work pace was dichotomized into low (≤ 3.8) and high (> 3.8).

#### Discrimination by regression model and decision tree

Discrimination between participants with and without mental LTSA during follow-up was evaluated by receiver operating characteristic curve (ROC) analysis, using the probabilities estimated by the prediction model and the decision tree. The area under the ROC-curve (AUC) represents discrimination between employees with and without mental LTSA during follow-up. AUC < 0.60 represents failing, 0.60–0.69 poor, 0.70–0.79 fair, 0.80–0.89 good, and 0.90–1.00 perfect discrimination. The AUCs were compared by using the non-parametric Wilcoxon statistic according to Hanley and McNeil (1982).

For the decision tree, risk groups were defined according to the development study. For the regression model, cut-off points set at 0.5, 1.0, 1.5, and 2.0 times the population mental LTSA risk were examined in more detail.

## Results

The 1736 occupational health survey participants had a mean age of 46.1 (standard deviation [SD] = 10.1) years; they worked on average 36.4 (SD = 7.3) hours per week as technicians (50%), office workers (43%), or shop assistants (7%). Table 1 shows the scores on the predictor variables of employees with and without mental LTSA during 1-year follow-up. Employees with mental LTSA had lower scores on learning opportunities, support from co-workers, support from family/friends and work satisfaction.

**Table 1:** Population characteristics of occupational health survey participants (*n* = 1736)

	Mental LTSA^a^ (*n* = 51)				No mental LTSA^a^ (*n* = 1685)				Analysis
	Mean	SD^b^	*n*	%	Mean	SD^b^	*n*	%	*p*
Sociodemographic variables									
Gender									0.18^c^
Men			29	57			1121	66	
Women			22	43			564	34	
Years employed at company	16.9	9.5			15.3	10.7			0.13^c^
Psychosocial work factors (range 1–5)									
Work pace	2.7	0.8			2.7	0.8			0.62^e^
Cognitive demands	3.5	0.7			3.6	0.8			0.36^e^
Role clarity	3.7	0.7			3.8	0.7			0.26^e^
Learning opportunities	2.7	1.1			3.0	0.9			0.03^e^
Support co-workers	3.4	0.9			3.9	0.8			0.00^e^
Social support family/friends (range 1–5)	3.3	1.1			3.6	1.0			0.01^e^
Work satisfaction (range 1–5)	3.7	0.8			4.0	0.8			0.00^e^
Distress									0.00^d^
Low			20	39			1183	70	
Medium			21	41			378	22	
High			10	20			124	8	

^a^Long-term sickness absence due to mental disorders

^b^Standard deviation

^c^Chi-square test

^d^Non-parametric Mann–Whitney *U* test

^e^Parametric Student’s *t* test

At 1-year follow-up, 51 (3%) participants had mental LTSA: *n* = 34 stress-related disorders, *n* = 8 depressive disorders, *n* = 4 anxiety disorders and *n* = 5 other psychiatric disorders.

### Validation of the prediction model

The regression model discriminated (AUC = 0.700; 95% CI 0.628–0.773) between participants with and without mental LTSA during follow-up. This implicates that for each random pair of participants, the prediction model correctly assigned the highest risk to the participant with mental LTSA during follow-up in 70.0% of the cases.

At a cut-off risk 0.5 times population risk, most participants (*n* = 47) with mental LTSA would be identified, at the cost of inviting 76% of all survey participants to preventive consultations (Table 2). At a cut-off risk 1.5 times the population risk, 17% of all survey participants would be invited for preventive consultations, but only 20 of 51 participants with mental LTSA would be identified.

**Table 2:** Cut-off points for risk of mental LTSA

Cut-off risk	Number^a^	%^a^	TP^b^	FP^c^	Sens^d^	Spec^e^	PPV^f^	NPV^g^
0.015	1316	76	47	1269	0.92	0.25	0.06	0.99
0.030	583	34	33	550	0.65	0.67	0.06	0.98
0.045	301	17	20	281	0.39	0.83	0.06	0.98
0.060	161	9	13	148	0.25	0.91	0.08	0.98

The table shows the number of occupational health survey participants at risk, as well as the number of true and fasle positives, sensitivity, specificity and (positive and negative) predictive values at cut-off risks 0.015 (half time population risk), 0.030 (population risk), 0.045 (1.5 times population risk) and 0.060 (2 times population risk)

^a^Number (%) of participants above cut-off risk

^b^Number of true positives

^c^Number of false positives

^d^Sensitivity

^e^Specificity

^f^Positive predictive value

^g^Negative predictive value

### Validation of the decision tree

**Fig. 1:**
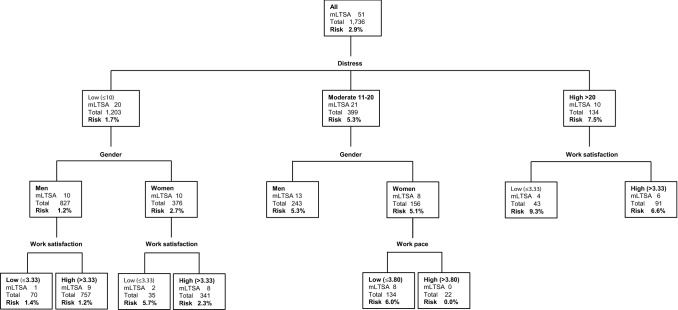
Decision tree (*n* = 1736)

The decision tree correctly assigned the highest risk of mental LTSA in 67.1% of the cases (AUC = 0.671; 95% CI 0.589–0.753). Although lower, the AUC did not differ significantly (*p* = 0.62) from that of the regression model. Survey participants with low, moderate and high distress scores had a 1.7%, 5.3% and 7.5% mental LTSA risk, respectively (Fig. 1). Among survey participants reporting low distress, there was no substantial gender difference in mental LTSA risk. Among women experiencing moderate distress, those reporting low work pace had a higher mental LTSA risk than those reporting high work pace.

## Discussion

The present study externally validated the ability of a previously developed prediction model and decision tree to identify occupational health survey participants at increased risk of mental LTSA in the year following the survey. Both the prediction model and the decision tree poorly discriminated between survey participants with and without mental LTSA at 1-year follow-up. Discrimination by the prediction model including nine predictor variables was not better than discrimination by a simpler decision tree based on distress, gender and work satisfaction.

It is easier for OHPs to use the decision tree because it readily shows the risk groups. Despite the ‘pruning’ in the development study, the decision tree was not stable. For example, women reporting moderate distress and low work pace had the highest risk of mental LTSA, whereas in the development study those with high work pace had the highest mental LTSA risk (van Hoffen et al. 2019). It should be noted that in the present study the number of women reporting high work pace was limited (*n* = 22). If the decision tree was re-estimated work pace would not partition the current study population. Only distress and work satisfaction would split the present population into risk groups [data not shown]. Although the decision tree requires further validation, we assume it will apply to new samples of occupational health survey participants because distress, gender and work satisfaction were significant splitting variables in both the development and the validation study.

## Strengths and weaknesses of the study

External validation studies are necessary to evaluate the generalization of risk predictions by prediction models and decision trees. The use of a new sample of occupational health survey participants is an asset of the present study since it provides insight whether predictions hold true in subjects working in a different setting and in a different time frame (Steyerberg and Harrell 2016). The prospective design and the use of recorded OP-certified mental LTSA are further strengths of the study.

Marital status was not available from the occupational health survey questionnaire. Consequently, it was not possible to externally validate the original regression model. Marital status was the least strong predictor in the development study (van Hoffen et al. 2019) and therefore it is unlikely that excluding marital status as predictor variable has substantially weakened the quality of the prediction model. The AUCs of the validated prediction model (AUC = 0.700) and decision tree (AUC = 0.671) were lower but of the same magnitude as those in the development study (AUC = 0.713 and AUC = 0.709, respectively). Although the prediction model and decision tree were internally validated at development, the poorer discrimination might still be indicative of over-optimistic predictions in the development study. Poorer discrimination may also have been caused by the fact that all participants in the present study worked in the same economic sector.

### Practical implications

Discrimination by the prediction model was better than discrimination by chance, but an AUC of 0.70 is not sufficient for using the prediction model in occupational healthcare practice, all the more because there is no optimal cut-off point to decide who is at increased risk of mental LTSA. For high-risk cut-off points, sensitivities are dangerously low, whereas for low-risk cut-off points the number of false-positives is approximately 20-fold the number of true positives. As a result, many survey participants may be incorrectly stigmatized as future mental health patients. Thus, the prediction model should not be used to identify survey participants for actions or measures aimed at preventing mental LTSA.

Nevertheless, mental LTSA risk estimates can be used to determine whether or not to address mental LTSA risk factors in the preventive OHP consultations after completing the occupational health survey questionnaire. For that purpose, the decision tree is more user-friendly than the prediction model, because it is easier to interpret and readily shows the mental LTSA risk groups. When consulting a survey participant who experiences low distress, the OHP may not be inclined to address mental LTSA risk factors in the preventive consultation. However, the decision tree showed that women reporting low distress levels have a two-fold risk of mental LTSA as compared to the total population when they experience low work satisfaction. In that case, it would important to explore mental LTSA risk factors, even if distress levels are low.

It is obvious to pay extra attention to mental LTSA risk factors in preventive OHP consultations when survey participants report high distress levels. Apart from addressing psychosocial stressors and coping strategies, it may be important to explore work satisfaction as the decision tree showed that survey participants with high distress and low work satisfaction have a three-fold higher mental LTSA risk than the population, as compared to a two-fold higher risk among those with high distress who are highly satisfied with their work.

At the moment, the decision tree is introduced in the OHS as a tool to target preventive consultations to mental LTSA risk factors if appropriate. It would be interesting to investigate if OHPs use the decision tree and whether or not this results in more actions (e.g., coaching by social workers or preventive treatment by psychologists) to prevent mental LTSA.
